# Genome sequence of an extremely halophilic archaeon isolated from Permian Period halite, Salado Formation in New Mexico, USA: *Halobacterium* sp. strain NMX12-1

**DOI:** 10.1128/mra.00778-24

**Published:** 2024-10-21

**Authors:** Leonardo Soto, Priya DasSarma, Brian P. Anton, Tamas Vincze, Ishita Verma, Bora Eralp, Dennis W. Powers, Brian Lee Dozier, Richard J. Roberts, Shiladitya DasSarma

**Affiliations:** 1 Marine Estuarine Environmental Sciences Program, University of Maryland, College Park, Maryland, USA; 2 Blue Marble Space Institute of Science, Seattle, Washington, USA; 3 Institute of Marine and Environmental Technology, University System of Maryland, Baltimore, Maryland, USA; 4 Department of Microbiology and Immunology, School of Medicine, University of Maryland, Baltimore, Maryland, USA; 5 New England Biolabs, Ipswich, Massachusetts, USA; 6 University of Mississippi, University, Mississippi, USA; 7 WIPP Test Coordination Office, Los Alamos National Laboratory (LANL), Carlsbad, New Mexico, USA; Montana State University, Bozeman, Montana, USA

**Keywords:** halophile, Haloarchaea, acidic proteome, transcription factors, extremophile, HOGs, DNA methylation, origin recognition complex, insertion sequence, rhodopsin

## Abstract

*Halobacterium* sp. strain NMX12-1, an extremely halophilic Archaeon, was isolated from 250 million-year-old Salado Formation salt crystal in Carlsbad, New Mexico. Single-molecule real-time sequencing revealed a 3.2-Mbp genome with a 2.6-Mbp chromosome and five plasmids (234, 211, 119, 21, and 1.6-kbp). The GC-rich genome encodes an acidic proteome, characteristic of Haloarchaea.

## ANNOUNCEMENT

The haloarchaeon, *Halobacterium* sp. strain NMX12-1, was isolated from late Permian Period (250 million years old) collected from 670-m-thick Salado Formation halite, 655 m below ground level (382 m above mean sea level) and ambient temperature of 26 °C, at the Waste Isolation Pilot Plant mine (32°22′18″N, 103°47′37″W) near Carlsbad, New Mexico on 6 November 2019 (1, 2). Isolates from ancient halite are a valuable resource for understanding the adaptation to extreme climate conditions present during the Permian Period.

Surface sterilized halite was dissolved in CM^+^ medium (complete medium plus trace metals), and growth was stimulated with shaking at 220 rpm at 37°C under illumination for 2 months (3). Enrichment cultures were plated on CM^+^ agar; candidate colonies were picked and grown in culture tubes with shaking for 1–2 weeks (New Brunswick Innova 4230, New Brunswick, NJ, USA), followed by streaking. After three rounds of purification, colonies appeared uniform, as previously described (4–9). Nucleic acids were extracted using the Monarch Genomic DNA Purification Kit T3010S (NEB).

Single-molecule real-time (SMRT) sequencing was performed using the PacBio Sequel platform (Menlo Park, CA, USA). A SMRTbell sequencing library was prepared from 1.7-µg genomic DNA at a mean fragment length of 14,116 bp, without further shearing, using SMRTbell Express Template Prep Kit 2.0. The library was sequenced on a single SMRT cell (Sequel Binding Kit 3.0, Sequel Sequencing Plate 3.0) with a 20-h collection time and no pre-extension. Sequencing reads were filtered and assembled *de novo* using the Microbial Assembly pipeline (SMRT Link 10.2.0.133434) with default parameters. A total of 2,380,522 subreads (94.5%) were aligned to the draft assembly, with a mean subread length of 7,432 bp and N50 subread length of 10,782 bp. The polished assembly rotated based on GC-skew distribution by the PacBio Microbial Assembly application automatically resolved into the large circular chromosome (2,614,549 bp), four circular plasmids (211,126; 119,415; 21,344, and 1,570 bp), and one additional contig (234,033 bp) with mean coverage of 2,163×.

Genome annotation was performed using the National Center for Biotechnology Information (NCBI) Prokaryotic Genome Annotation Pipeline build 3190, and taxonomy was determined by NCBI (10–12). The GC-rich (66.5%) genome encodes 3,369 proteins with a calculated mean isoelectric point of 4.7 (highly acidic), characteristic of Haloarchaea (13). The genome includes a single rRNA operon and 60 tRNA genes based on tRNAscan-SE 2.0, with 3 containing introns (Ile2, Trp, and Met) (14). Based on GeneMark.hmm 2, EMBOSS (version 6.6.0.0) analysis, HaloWeb tools, and BLAST 2.11.0, genes were found coding for 795 out of 799 conserved core haloarchaeal groups and all 83 haloarchaeal signature groups, including 6 Orc/Cdc6 proteins, 5 TATA-binding proteins, 11 TFIIB proteins, and a single sensory (SRII) rhodopsin. In addition, 10 haloarchaeal insertion sequence (ISH) elements were present, including 6 ISH3s, 2 ISH8s, an ISH9, and an ISH12 (15–28).

Methylation patterns were determined using the base modiﬁcation and motif analysis application within the same SMRT Link environment using default settings (minimum Qmodscore = 100). The following methylated DNA motifs were identiﬁed, with underlined bases representing m4C or m6A, or their complements: CTAG (ABSL23_14290), GACGTC (ABSL23_01420), CTCGAG (ABSL23_15640), CATCCG (unassigned), CACATC (unassigned), TACGAG (unassigned), and CAYNNNNNGTCA (ABSL23_03615), deposited in REBASE (28). For a survey of archaeal methylation systems, see reference (29).

## Data Availability

The *Halobacterium* sp. NMX12-1 (BioProject PRJNA412908, BioSample SAMN41842361) genome sequence was deposited at GenBank (CP159204: chromosome, CP159203: pNMX12-1_234, CP159205: pNMX12-1_211, CP159206: pNMX12-1_119, CP159207: pNMX12-1_21, and CP159208: pNMX12-1_2) and raw data in the NCBI Sequence Read Archive (SRR29517185).

## AFTER EPUB

*[This article was published on 21 October 2024 with an error in the second paragraph. A missing word was added in the current version, posted on 25 October 2024.]*
